# Revamping hemothorax management: The promise of low‐dose intrapleural fibrinolytic therapy as an alternative

**DOI:** 10.1002/rcr2.70012

**Published:** 2024-08-26

**Authors:** Mas Fazlin Mohamad Jailaini, Yusra Hashim, Mohamed Faisal Abdul Hamid

**Affiliations:** ^1^ Respiratory Unit, Faculty of Medicine Universiti Kebangsaan Malaysia (UKM) Kuala Lumpur Malaysia

**Keywords:** alteplase, hemothorax, intrapleural fibrinolysis

## Abstract

Surgical evacuation has long been the standard treatment for hemothorax. However, some patients are not suitable candidates for surgery. Intrapleural fibrinolytic therapy (IPFT) has recently emerged as an effective alternative for managing retained hemothorax. This case report describes two patients with retained hemothorax who were unfit for surgery and were successfully treated with IPFT at our centre. Both patients were deemed unsuitable for surgery due to comorbidities and their overall functional status. They received three cycles of IPFT, each consisting of 2.5 mg of alteplase. This treatment effectively evacuated the retained hemothorax, achieving complete radiological resolution without immediate or delayed complications up to 3 months post‐discharge.

## Graphical abstract

The use of intrapleural alteplase is well established in pleural empyema, however, no large randomized trial available on the use of this agent for retained hemothorax. This case report explores the rationale behind using intrapleural Alteplase as an alternative to surgery in the management of retained hemothorax

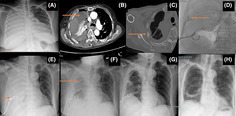

## INTRODUCTION

Hemothorax is defined as the accumulation of blood in the pleural space, characterized by a pleural fluid‐to‐serum haematocrit ratio exceeding 50%.^1^ It is commonly associated with traumatic injury to thoracic anatomical structures.^2^ The typical mechanisms of trauma include blunt or penetrating injuries to intrathoracic and extrathoracic structures, resulting in bleeding into the pleural space. Non‐traumatic causes of hemothorax are less common and include iatrogenic factors, neoplasms, coagulopathies, and infections.^3^

Treatment of hemothorax depends on the volume of blood collected in the pleural space. Minimal collections, defined as less than 300 mL, typically do not require intervention and usually necessitate no management.^4^ In contrast, collections exceeding 1000 mL are considered massive hemothorax and require definitive intervention to halt ongoing haemorrhage and evacuate the pleural space.^4^ When the size and severity of hemothorax warrant intervention, tube thoracostomy remains the gold standard treatment in most medical centres.^5^ Management options vary on a case‐by‐case basis and traditionally include observation, percutaneous drainage, or surgery. However, there is growing interest among pulmonologists in using intrapleural fibrinolytic therapy (IPFT) to manage hemothorax, with numerous studies and case reports demonstrating successful outcomes.

In this case series, we report two patients with retained hemothorax, unfit for surgery, who were successfully treated with IPFT at our centres.

## CASE REPORT

### Case 1

**FIGURE 1:**
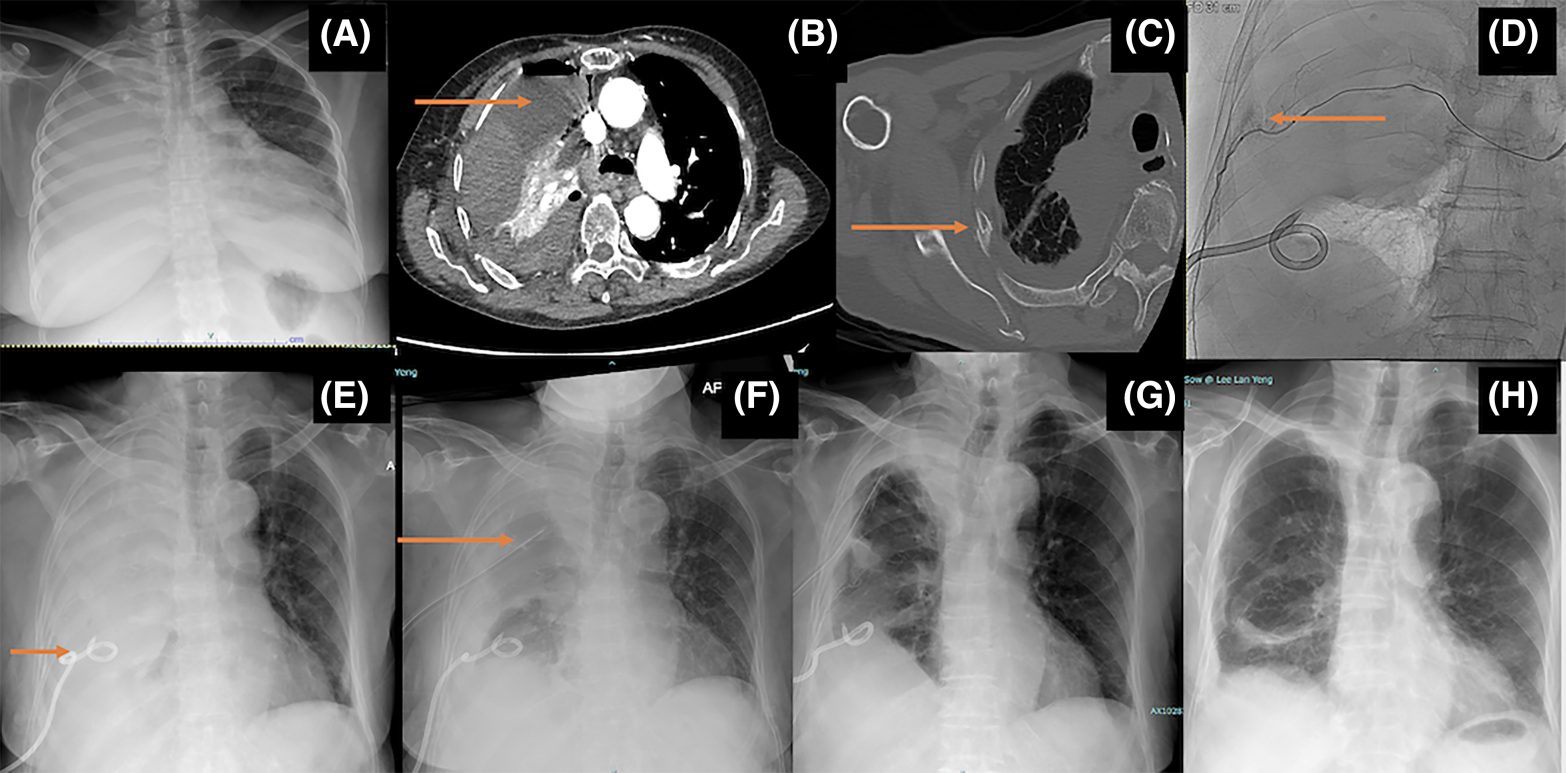
Chest radiograph (A) on arrival revealed massive right pleural effusion. Four contrast enhanced computed tomography (CECT) Thorax (B) revealed heterogenous hyperdensity of pleural fluid (red arrow) consistent with hemothorax. CECT Thorax (C) revealed right 7th rib fracture. CT Thoracic angiogram (D) revealed bleeding right 7th intercostal artery. Chest radiograph (E) revealed ICC in‐situ (red arrow) with persistent hemothorax. Chest radiograph (F) post large bore chest tube (red arrow) showed slight reduction of right hemithorax opacity. Chest radiograph (G) post 4 cycles of intrapleural alteplase showed significant radiological improvement. Chest radiograph (H) one month post discharge revealed resolution of hemothorax.

An 82‐year‐old woman with a history of hypertension and ischemic heart disease (IHD); on aspirin, who had frequent falls at home, presented with 1 day history of dyspnea. Upon arrival at the emergency department, she was tachypneic with a respiratory rate of 30 breaths per minute, and blood gases indicated type 1 respiratory failure. A chest radiograph (Figure 1A) revealed a moderate right pleural effusion.

Right thoracentesis done, aspirating frank blood. Pleural fluid cytology was negative for malignancy and culture was sterile. Pleural fluid haematocrit was not sent as this test was not analysed by our lab. An immediate contrast enhanced computed tomography (CECT) thorax revealed heterogenous hyperdensity effusion consistent with hemothorax (Figure 1B) and 7th rib fracture (Figure 1C). CT thoracic angiogram revealed a bleeding right 7th intercostal artery (Figure 1D), which was successfully embolized. There was an initial drop in haemoglobin from 10.5 to 9.2 g/dL over the first 24 h of admission, but it remained stable following embolization. One pint of packed cells was transfused.

Right intercostal chest catheter (ICC) 12Fr inserted post‐embolization; however there was persistent right effusion; (Figure 1E) subsequently the next day, a large‐bore (24 F) chest tube was inserted under ultrasound guidance (Figure 1F). Despite referral to the cardiothoracic team, it was decided, after discussion, that due to the patient's advanced age and frail condition, non‐surgical management was preferable. The total drainage recorded with the large‐bore chest tube was 2.5 L.

However, the chest radiograph showed a persistent effusion, and the chest tube drainage was inadequate. Therefore, IPFT (alteplase) was administered 5 days following large bore chest tube insertion. Alteplase was reconstituted in 50 cc of normal saline, allowed to dwell for 45 min, and then the tube was unclamped to release the effusions. Marked improvement was observed after 4 cycles of alteplase, with no adverse events. A total of 2 L of retained hemothorax was drained after four cycles of IPFT with alteplase. Both clinical and radiological improvements (Figure 1G) were noted, and the patient was subsequently discharged. Follow‐up in the clinic a month later showed resolution of the hemothorax (Figure 1H).

### Case 2

**FIGURE 2:**
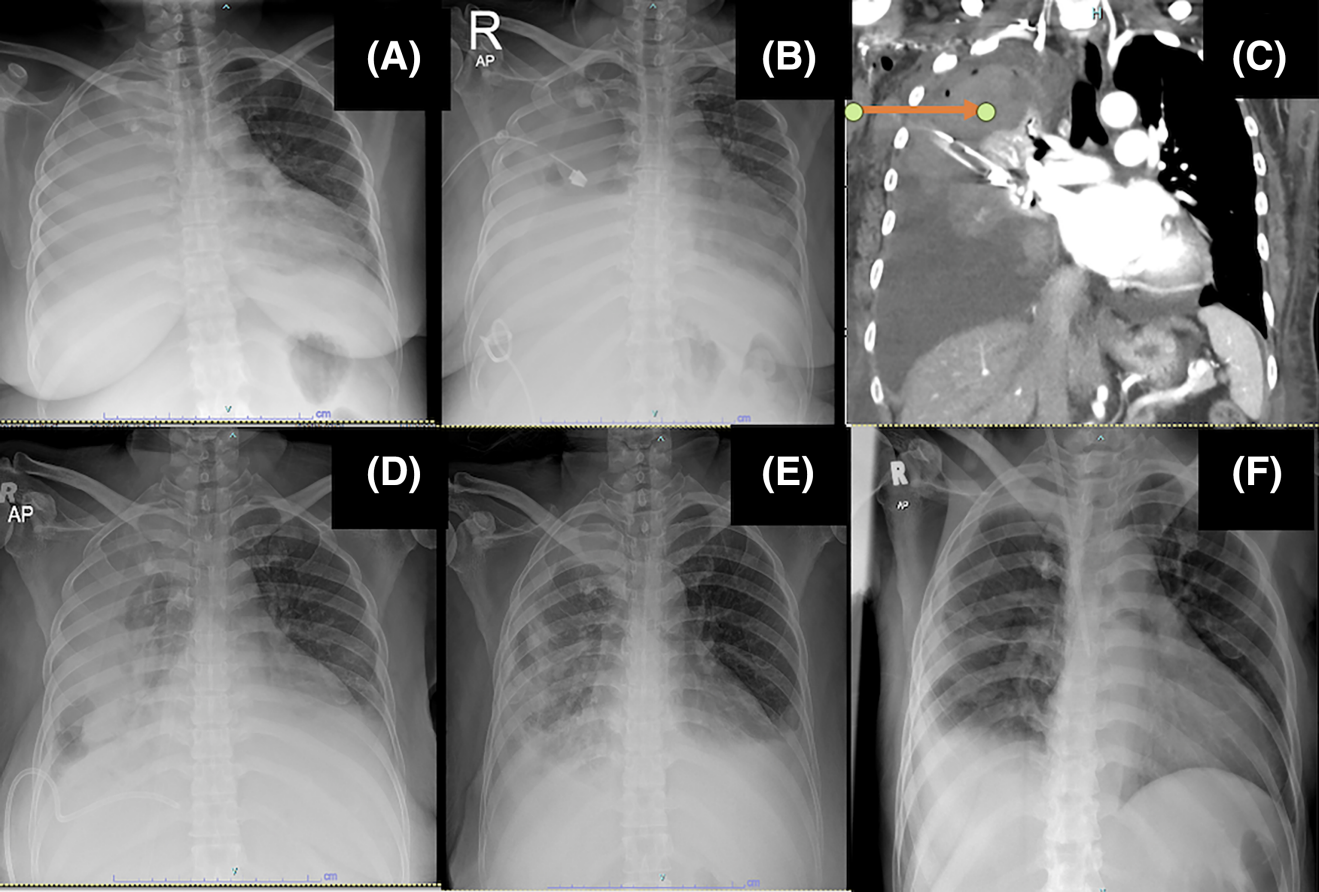
Chest radiograph (A) on arrival revealed massive right pleural effusion. Chest radiograph (B) post large post chest tube. Revealed persistent hemothorax. Contrast enhanced computed tomography thorax (C) revealed hyperdensity in the pleural fluid consistent with hemothorax. Chest radiograph (D) post 2 doses of IPFT showed radiological improvement. Chest radiograph (E) post 6 doses of IPFT showed further improvement of right hemithorax opacity. Chest radiograph (F) 1 month post discharge showed resolution of hemothorax.

A 50‐year‐old woman with a history of diabetes mellitus, hypertension, advanced kidney disease, and IHD (on clopidogrel) presented with a 3‐week history of dyspnea. She had no history of trauma. On examination, she was tachypneic with a respiratory rate of 30 breaths per minute and an oxygen saturation of 60% on room air. A chest radiograph upon arrival revealed total massive right pleural effusion (Figure 2A).

A large‐bore chest tube (32 F) was inserted (Figure 2B), draining 600 cc of hemorrhagic fluid. The patient was then sent for a CT thoracic angiogram, which revealed a small pseudoaneurysm with hemopneumothorax (Figure 2C). However, no active arterial bleeding or lung parenchymal injury was detected in the right intrathoracic region. Further pleural fluid analysis revealed no malignant cell was detected on cytology and no organism detected on pleural fluid culture. There was a slight drop in haemoglobin from 10.1 to 9.5 g/dL, but it remained stable thereafter. No blood transfusion was required.

The drainage was inadequate, and the patient remained symptomatic. A referral to the cardiothoracic surgery team was made; however, due to the patient's pre‐existing conditions, she was deemed unfit for surgery. Consequently, it was decided to opt for medical therapy to manage the retained hemothorax. The chest tube was removed as it was not functioning, and another intercostal catheter (pigtail) was inserted 2 days later. However, there was no clinical or radiological improvement.

A bedside ultrasound of the thorax revealed multiple septations and loculations interfering with the drainage of the hemothorax. Consequently, the patient was selected for intrapleural fibrinolytic therapy (IPFT) which was given at day 6 of admission. Intrapleural alteplase, 2.5 mg reconstituted in 50 cc normal saline, was administered. Following two doses of intrapleural alteplase (given 12 h apart); there was significant radiological improvement (Figure 2D). After six cycles of IPFT, a total of 3 L of hemorrhagic fluid was drained. The patient showed marked symptomatic and radiological improvement (Figure 2E) and was able to maintain oxygen saturation on room air. She was subsequently discharged, and a follow‐up chest radiograph a month later (Figure 2F) showed a resolution of the hemothorax.

## DISCUSSION

Retained hemothorax has been the most common sequelae of hemothorax and has been estimated to occur in almost 40% of cases with traumatic or non‐traumatic hemothorax.^6^ It is defined as residual hemothorax in the pleural cavity measuring more than 500 mL or hemothorax that occupies more than one‐third of the thoracic cavity and any residual blood that cannot be drained out after 72 h of tube thoracostomy insertion.^7^ Once retained hemothorax is suspected, the residual blood needs to be evacuated to prevent further progression into fibrothorax.

Guidelines recommend surgical intervention such as video‐assisted thoracoscopic surgery (VATS) in treating hemothorax or sequelae of hemothorax. However, as in our case series; some patients are at high risk of undergoing this procedure due to underlying comorbidities. Both patients has an American Society of Anaesthesiologists physical status classification of III which carries mortality up to 25.9%.^8^

IPFT has been showing promising results in the management of pleural diseases.^9^ However, the usage of IPFT remains controversial as not many studies or research are being conducted to prove the safety and efficacy of IPFT in managing retained hemothorax. A study by Kumar et al. using intrapleural streptokinase demonstrated comparable effectiveness in resolving retained hemothorax to VATS, with similar rates of morbidity.^10^ A meta‐analysis by Hendriksen et al. showed IPFT could reduce the need for operative intervention in trauma patients with retained traumatic hemothorax. Hence, the evidence suggests the beneficial effect of IPFT in patients with retained hemothorax who may not be candidates for surgery.^11^

In this case series, we demonstrated successful IPFT done in two different cases of retained hemothorax as an alternative to surgery; in adult patients who had been hospitalized because of massive hemothorax. This case series particularly demonstrated the use of alteplase as the fibrinolytic agent.

Alteplase facilitate the breakdown of the fibrins and alteplase will also stimulate intrapleural fluid accumulation, which in turn provides an intrapleural lavage effect that will improve the evacuation of retained and remnants of collections.^12^, ^13^ This will eventually lead to lung expansion and, in turn, will subsequently lead to adequate pleural appositions, which are the effect needed and crucial in achieving successful pleurodesis. As there is no evidence of pleural infection, a combination with deoxyribonuclease (DNAse) is not needed.

In our center, the IPFT protocol was designed mostly for the treatment of complex pleural effusions, lung empyema, or malignant pleural effusions. The same protocol was applied to treat retained hemothorax (without DNAse), as discussed in this case series.

The risk of bleeding after IPFT has always been a major concern for clinicians; however, it rarely occurs. Akulian et al. reported a pleural bleeding occurrence of only 4.1%.^14^ Increased rates of pleural bleeding are associated with the concurrent use of anticoagulation, but this risk can be reduced by withholding anticoagulation before IPFT.^14^

Serial haemoglobin trends should be monitored during therapy to watch out for immediate and delayed side effects of the procedure. In both of our patients, there was no bleeding post‐IPFT.

In summary, for decades, the crux of management in treating retained hemothorax has been in favour of surgical evacuation. However, low‐dose IPFT provided symptomatic and radiological resolution in selected patients, suggesting it as a viable alternative treatment option. Decisions should be made by a multidisciplinary team, including respiratory specialists, thoracic surgeons, and interventional radiologists. The findings from our case series may serve as a basis for future larger prospective studies.

## AUTHOR CONTRIBUTIONS

Mas Fazlin Mohamad Jailani and Mohamed Faisal Abdul Hamid were involved with the concept, acquisition of data and drafting of manuscript. Yusra Hashim was involved with literature review. Mohamed Faisal Abdul Hamid contributed to critical revision of important intellectual content. All authors had full access to data, contributed to the paper, approved the final revision for publication and take responsibility for its accuracy and integrity.

## CONFLICT OF INTEREST STATEMENT

None declared.

## ETHICS STATEMENT

The authors declare that appropriate written informed consent was obtained for the publication of this manuscript and accompanying images.

## Data Availability

The data that support the findings of this study are available from the corresponding author upon reasonable request.
